# Characterization of *Sinomonas gamaensis* sp. nov., a Novel Soil Bacterium with Antifungal Activity against *Exserohilum turcicum*

**DOI:** 10.3390/microorganisms7060170

**Published:** 2019-06-08

**Authors:** Yansong Fu, Rui Yan, Dongli Liu, Junwei Zhao, Jia Song, Xiangjing Wang, Lin Cui, Ji Zhang, Wensheng Xiang

**Affiliations:** 1 Heilongjiang Provincial Key Laboratory of Agricultural Microbiology, Northeast Agricultural University, Harbin 150030, China; yansongfu1@163.com (Y.F.); 15546073308@163.com (R.Y.); 17862181612@163.com (D.L.); guyan2080@126.com (J.Z.); songjia-hit@hotmail.com (J.S.); wangneau2013@163.com (X.W.); 15663843306@163.com (L.C.); 2 State Key Laboratory for Biology of Plant Diseases and Insect Pests, Institute of Plant Protection, Chinese Academy of Agricultural Sciences, Beijing 100193, China

**Keywords:** novel species, *Sinomonas*, antifungal activity, *Exserohilum turcicum*

## Abstract

A novel Gram staining positive, aerobic bacterium NEAU-HV1^T^ that exhibits antifungal activity against *Exserohilum turcicum* was isolated from a soil collected from Gama, Hadjer lamis, Chad. It was grown at 10–45 °C (optimum 30 °C), pH 5–10 (optimum pH 8), and 0–4% (w/v) NaCl (optimum 1%). Phylogenetic analysis based on 16S rRNA gene sequences showed that strain NEAU-HV1^T^ was closely related to *Sinomonas susongensis* A31^T^ (99.24% sequence similarity), *Sinomonas humi* MUSC 117^T^ (98.76%), and *Sinomonas albida* LC13^T^ (98.68%). The average nucleotide identity values between NEAU-HV1^T^ and its most closely related species were 79.34−85.49%. The digital DNA–DNA hybridization values between NEAU-HV1^T^ and *S. susongensis* A31^T^, *S. albida* LC13^T^, and *S. humi* MUSC 117^T^ were 23.20, 23.50, and 22.80%, respectively, again indicating that they belonged to different taxa. The genomic DNA G+C content was 67.64 mol%. The whole cell sugars contained galactose, mannose, and rhamnose. The polar lipids were diphosphatidylglycerol, phosphatidylglycerol, phosphatidylinositol, and four glycolipids. The respiratory quinone system comprised MK-9(H_2_), MK-10(H_2_), and MK-8(H_2_). The major cellular fatty acids (>5%) were *anteiso*-C_15:0_, *anteiso*-C_17:0_, C_16:0_, and *iso*-C_15:0_. Based on the polyphasic analysis, it is suggested that the strain NEAU-HV1^T^ represents a novel species of the genus *Sinomonas*, for which the name *Sinomonas gamaensis* sp. nov. is proposed. The type strain is NEAU-HV1^T^ (= DSM 104514^T^ = CCTCC M 2017246^T^).

## 1. Introduction

The genus *Sinomonas*, a member of the family *Micrococcaceae* in the phylum *Actinobacteria*, was first proposed by Zhou et al. [1] with the description of *Sinomonas flava* and the reclassification of *Arthrobacter atrocyaneus* as *Sinomonas atrocyaneus*. At the time of writing, the genus *Sinomonas* comprised 10 species (http://www.bacterio.net/sinomonas.html) that were isolated from soil, volcanic rock, and the surface of weathered biotite: *Sinomonas flava*, *Sinomonas atrocyaneus*, *Sinomonas echigonensis*, *Sinomonas albida*, *Sinomonas soli*, *Sinomonas notoginsengisoli*, *Sinomonas mesophila*, *Sinomonas susongensis*, *Sinomonas humi*, and *Sinomonas halotolerans* [1,2,3,4,5,6,7]. The species of this genus are aerobic and rod-shaped, contain MK-9(H_2_) as the predominant respiratory quinone, and have *iso*-C_15:0_, *anteiso*-C_15:0_, and *anteiso*-C_17:0_ as the major fatty acids. Other typical features are the presence of galactose, mannose, and ribose as the major cell wall sugars; A3α as the peptidoglycan type; and diphosphatidylglycerol, phosphatidylglycerol, and phosphatidylinositol as the major phospholipids. Some members of the genus *Sinomonas* have been reported to be able to synthesize silver nanoparticles with antimicrobial activity [8], hydrolyze starch [9], biodesulfurize coal [10], and degrade sesamin [11]. Recently, *S. atrocyaneus* has exhibited plant growth promoting effects and antagonistic activity against a broad-spectrum of root and foliar pathogens, implying its potential use in sustainable agriculture [12]. In this study, a strain NEAU-HV1^T^ with inhibitory activity against phytopathogenic fungi *Exserohilum turcicum* was isolated from a soil sample. Based on the polyphasic analysis, this strain was classified as representative of a novel species in the genus *Sinomonas*.

## 2. Materials and Methods

### 2.1. Isolation of Bacterial Strain

Strain NEAU-HV1^T^ was isolated from a soil sample collected from a cotton field (13°0´N, 15°44´E), situated in Gama, Hadjer lamis Region, Chad, which is considered as the principal agricultural region of Chad. The soil sample was suspended in sterile water (2 mL) followed by an ultrasonic treatment (160 w) for 3 min. After the addition of distilled water (43 mL), the soil suspension was incubated at 28 °C and 250 rpm on a rotary shaker for 20 min. Then, 200 µL of the suspension was spread on a plate of humic acid-vitamin (HV) agar [13] supplemented with cycloheximide (50 mg L^−1^) to inhibit any fungal contaminants. After seven days of aerobic incubation at 28 °C, colonies were transferred and purified on nutrition agar (NA; BD Difco, Becton, Dickinson and Company, Sparks, MD, USA) and maintained as glycerol suspensions (20%, v/v) at −80 °C for long term preservation.

### 2.2. Morphological and Biochemical Characteristics of NEAU-HV1^T^

Colony morphology of strain NEAU-HV1^T^ was observed after two days of incubation at 28 °C on NA. Cell morphology was examined by transmission electron microscopy (Hitachi H-7650, Hitachi Co., Tokyo, Japan). Motility was detected by the presence of turbidity throughout tubes containing semi-solid medium and was further confirmed by the hanging-drop method [14,15]. Gram staining was performed as described by Smibert and Krieg [16]. Growth at different temperatures (0–45 °C, steps of 1 °C below 5 °C, and then intervals of 5 °C) was determined in a tryptic soy broth (TSB; BD Difco, Becton, Dickinson and Company, Sparks, MD, USA) medium after incubation for seven days. Growth tests for pH ranging from 4.0–12.0 in 1.0 pH unit intervals and NaCl tolerance (0–10% (w/v) in 1% intervals) were determined in TSB medium at 28 °C for seven days on a rotary shaker. The buffer systems were: pH 4.0–5.0, 0.1 M citric acid/0.1 M sodium citrate; pH 6.0–8.0, 0.1 M KH_2_PO_4_/0.1 M NaOH; pH 9.0–10.0, 0.1 M NaHCO_3_/0.1 M Na_2_CO_3_; and pH 11.0–12.0, 0.2 M KH_2_PO_4_/0.1 M NaOH [17]. Growth was determined by monitoring the turbidity at OD_600_ by using a spectroscopic method (U-3900; Hitachi Co., Tokyo, Japan) and plate counting. The utilization of organic compounds as the sole carbon and energy source, decomposition of cellulose, hydrolysis of starch and aesculin, reduction of nitrate, peptonization of milk, liquefaction of gelatin, and production of H_2_S were analyzed as recommended by Ventosa et al. [18]. Anaerobic growth was tested on NA for up to two weeks at 28 °C in Bacteron anaerobic chambers (Sheldon Manufacturing, Carson, CA). The methyl red and Voges–Proskauer reactions, hydrolysis of Tweens 20, 40, and 80, production of esterase and urease were tested as described by Smibert and Krieg [16]. Metabolic properties and enzyme activities were tested using API 50CH, API 20NE, and API ZYM strips with API 50CHB suspension medium (bioMérieux, Marcy-I’Etoile, France) according to the manufacturer’s protocols.

### 2.3. Phylogenetic Analysis of NEAU-HV1

For DNA extraction, strain NEAU-HV1^T^ was cultured in TSB to the early stationary phase (~24 hours) and harvested by centrifugation. The genomic DNA was extracted using a TIANamp Bacteria DNA Kit (TIANGEN Biotech, Co. Ltd., Beijing, China). The 16S rRNA gene was PCR-amplified with the universal primers 27F (5´-AGAGTTTGATCCTGGCTCAG-3´) and 1541R (5´-AAGGAGGTGATCCAGCC-3´) under conditions described previously [19]. The PCR product was purified and cloned into the vector pMD19-T (Takara) and sequenced using an Applied Biosystems DNA sequencer (model 3730XL, Applied Biosystems Inc., Foster City, California, USA) and software provided by the manufacturer. The almost full-length 16S rRNA gene sequence was compared with the available 16S rRNA gene sequences of validly named species in the EzBioCloud server (https://www.ezbiocloud.net/) [20] to search the related similar species for tree reconstructions and calculate the pairwise 16S rRNA gene sequence similarity. Phylogenetic trees were reconstructed with the neighbor joining [21], maximum likelihood [22], and minimum evolution [23] methods using MEGA version 7.0 after multiple alignments of the sequences using the CLUSTAL W algorithm [24]. For the neighbor joining algorithm, Kimuraʼs 2-parameter model and pairwise deletion were used to reconstruct the phylogenetic tree. The Tamura–Nei model together with Gamma distribution and Invariant sites (TN93+G+I) and Nearest-Neighbor-Interchange (NNI) heuristic method for the maximum likelihood algorithm, and close-neighbor-interchange (CNI) search for the minimum evolution algorithm were applied. Distances were calculated according to Kimura’s two-parameter model [25]. The topology of the phylogenetic tree was evaluated by using bootstrap analysis with 1000 replicates [26]. All positions containing gaps and missing data were eliminated from the dataset and the root position of the trees was inferred by using *Nocardia inohanensis* DSM 44667^T^ (GenBank accession no. AB092560) as an outgroup. The genome sequences of strain NEAU-HV1^T^ and its most closely related strains *S. susongensis* A31^T^, *S. albida* LC13^T^, and *S. humi* MUSC 117^T^ were determined by Novogene Bioinformatics Technology (Beijing, China) using a IlluminaHiSeq PE150 (Illumina) following the manufacturer’s suggested protocols. The resulting reads were quality trimmed to the Q20 confidence level. The draft genome was assembled using SOAP de novo version 2.04 [27] with default parameters. The estimated dDDH values were calculated using formula two at the Genome-to-Genome Distance Calculation (GGDC) website (http://ggdc.dsmz.de/distcalc2.php) as described by Meier-Kolthoff et al. [28]. Average nucleotide identity (ANI) calculations were performed using the OrthoANIu tool (https://www.ezbiocloud.net/tools/ani) [29] and JSpecies software (http://jspecies.ribohost.com/jspeciesws/) [30]. For core-genome analysis, cores genes were extracted from the genomes of NEAU-HV1^T^ and its related strains using the USEARCH program with BPGA (Bacterial Pan Genome Analysis tool), with >50% sequence identity cut-off [31]. The concatenated amino acid sequences of core genes were aligned to generate the phylogenetic tree using the neighbor joining method. Genome mining for bioactive secondary metabolites was performed using “antibiotics and secondary metabolite analysis shell” (antiSMASH) version 4.0 [32].

### 2.4. Chemotaxonomic Analysis of NEAU-HV1^T^

Biomass for chemical studies was prepared by growing the strains in TSB medium in shake flasks at 28 °C until the exponential phase of growth was reached. Cells were harvested by centrifugation, washed with distilled water, and freeze-dried. The cell-wall diamino acid was determined from whole-cell hydrolysates, as described by McKerrow et al. [33] and analyzed by a HPLC method using an Agilent TC-C_18_ column (250 × 4.6 mm, i.d. 5 μm) (Agilent Technologies, Santa Clara, CA, USA). Procedures for identification of cell-wall amino acids and whole-cell sugars were performed as described by Tang et al. [34]. Polar lipids were extracted and examined by two-dimensional TLC and identified according to the method of Minnikin et al. [35]. Menaquinones were extracted from freeze-dried biomass and purified according to Collins [36]. Extracts were analyzed using an HPLC-UV method [37]. Fatty acid methyl esters were extracted and analyzed using the microbial identification system (MIDI) and the RTSBA6 database (Microbial Identification Inc., Newark, DE, USA) [38].

### 2.5. In Vitro Antifungal Activity Test

The antagonistic activity of NEAU-HV1^T^ against six phytopathogenic fungi (*Fusarium oxysporum*, *Fusarium orthoceras*, *Sclerotinia sclerotiorum*, *Alternaria solani*, *Corynespora cassiicola*, and *Exserohilum turcicum*) was evaluated using petri dish assays [39]. To further investigate the antifungal components produced by NEAU-HV1^T^, this strain was cultured in GYM medium [40] and the inhibitory activity of the supernatant was tested. Briefly, strain NEAU-HV1^T^ was inoculated into GYM medium and incubated at 28 °C for seven days on a rotary shaker. The supernatant was obtained by centrifugation at 8000 rpm and 4 °C for 10 min and subsequently filtrated with a 0.2 μm membrane filter. The cell precipitate was extracted with an equal volume of methanol. Then, the antifungal activity was evaluated using the well diffusion method [41], and each well contained 150 μL of the supernatant or methanol extract. In order to test the effect of pH on the antifungal stability, pH values of the supernatant was adjusted to pH 3.0, 5.0, 7.0, and 11.0 by 1 mol/L hydrochloric acid or 1 mol/L NaOH, and the adjusted supernatant was kept at 4 °C for 24 h. Afterward, the pH values were readjusted to 7.0. To examine the effect of temperature on antifungal activity, the supernatant was placed in a water bath at 40, 60, 80, and 100 °C for 30 min, and then cooled to room temperature. A total of 200 μL of the treated supernatant was added into 25 mL of sterile potato dextrose agar (PDA), and the mixture was poured into 9 cm plates. The PDA containing 200 μL of sterilized water was used as the control. Then, 5 mm plugs of *E. turcicum* were placed at the center of each plate, and all plates were placed at 28 °C for seven days. The antifungal activity was evaluated when the fungal mycelium reached the edges of the control plates.

## 3. Results and Discussion

### 3.1. Polyphasic Taxonomic Characterization of NEAU-HV1^T^

**Figure 1:**
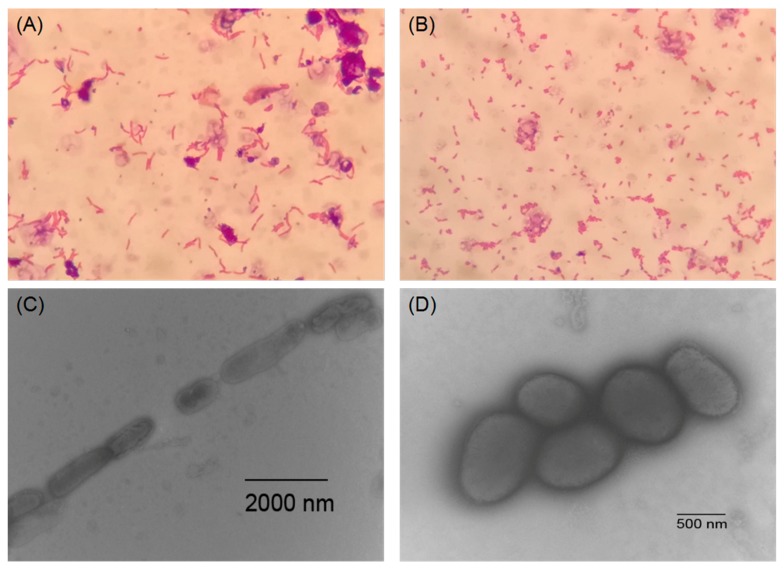
The morphological characteristics of strain NEAU-HV1^T^ on NA agar. (**A**,**B**) represent the light micrographs of Gram-staining cells of strain NEAU-HV1^T^ after incubation for 12 and 24 h, respectively. (**C**,**D**) represent the transmission electron micrograph of negatively staining cells of strain NEAU-HV1^T^ after incubation for 12 and 24 h, respectively.

Strain NEAU-HV1^T^ was aerobic, Gram staining positive, and non-motile. Light microscopy at 1000× magnification showed that the cells formed bent rods after cultivation for 12 h and then fragmented into a coccoid shape at 24 h, and the changed shape was also observed by transmission electron microscopy (Figure 1). This metamorphic feature of rod-coccus growth cycle was also observed for other species of *Sinomonas* such as *S. susongensis* A31^T^, *S. halotolerans* CFH S0499^T^, *S. mesophila* MPKL 26^T^, and *S. echigonensis* CW59^T^ [2,4,5,6]. Neither flagella nor spore were found. Colonies on NA were circular, convex, and pale yellow, with a smooth surface and diameter of 0.5–1.5 mm after incubation for 48 h at 28 °C. Strain NEAU-HV1^T^ was able to grow at 10–45 °C (optimum, 30 °C), at pH 5–10 (optimum, pH 8), and with 0–4% (w/v) NaCl (optimum, 1%). Phenotypic properties useful for distinguishing NEAU-HV1^T^ from its three phylogenetic neighbors are shown in Table 1 and Table S1. The detailed characteristics of strain NEAU-HV1^T^ are given in the species description.

**Figure 2:**
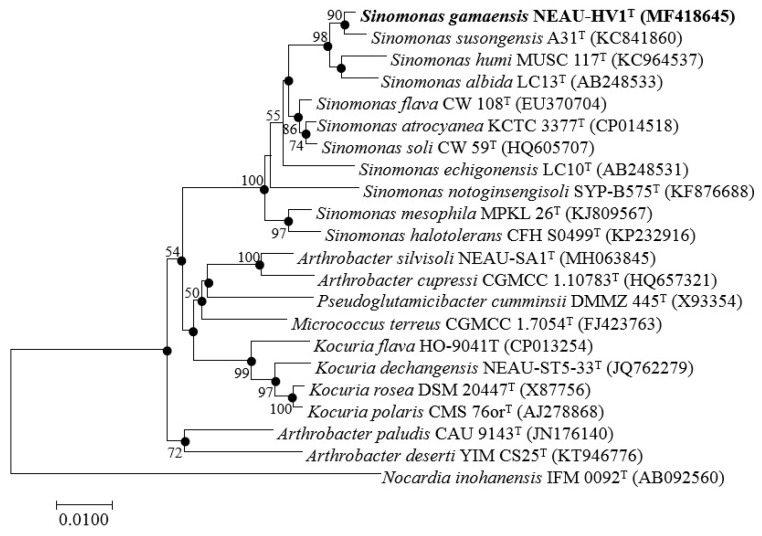
Neighbor joining tree showing the phylogenetic position of strain NEAU-HV1^T^ (1518 bp) and related taxa based on 16S rRNA gene sequences. Filled circles indicate branches that were also recovered using the maximum-likelihood and minimum evolution methods. Bootstrap values >50% (based on 1000 replications) are shown at branch points. Bar, 0.01 substitutions per nucleotide position.

**Figure 3:**
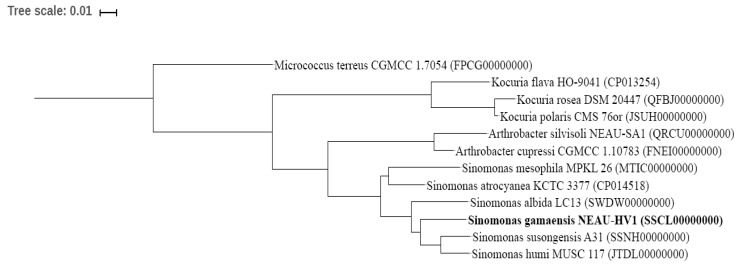
Neighbor joining tree showing the phylogenetic position of strain NEAU-HV1^T^ relative to closely related type strains based on the core genome.

BLAST sequence analysis of the 16S rRNA gene sequence (1518 bp; GenBank/EMBL/DDBJ accession number MF418645) indicated that strain NEAU-HV1^T^ was related to members of the genus *Sinomonas*. The EzBioCloud analysis showed that strain NEAU-HV1^T^ was most closely related to *S. susongensis* A31^T^, *S. humi* MUSC 117^T^, and *S. albida* LC13^T^ with a gene sequence similarity of 99.24, 98.76, and 98.68%, respectively. In the neighbor joining tree (Figure 2), strain NEAU-HV1^T^ clustered with members of the genus *Sinomonas* and formed an independent subclade with the most closely related strains of *S. susongensis* A31^T^, *S. albida* LC13^T^, and *S. humi* MUSC 117^T^. The topology of the neighbor joining tree was further confirmed using the maximum likelihood and minimum evolution methods (Figures S1 and S2). De novo assembly of the genome sequencing data of strain NEAU-HV1^T^ resulted in 39 contigs, an N50 of 233,829 bp, and an average genome coverage of 441.0× (Table 2). The draft genome size of strain NEAU-HV1^T^ was 4,320,429 bp and its DNA G+C content was 67.64 mol% for strain NEAU-HV1^T^, which is within the range (66.6–71.8 mol%) previously obtained for species of the genus *Sinomonas* [5]. A phylogenetic tree (Figure 3) based on the conserved core genome showed that NEAU-HV1^T^ was most related to *S. susongensis* A31^T^, *S. albida* LC13^T^, and *S. humi* MUSC 117^T^, supporting the phylogenetic analysis based on 16S rRNA gene sequences (Figure 2). The dDDH values (Table 2) between strain NEAU-HV1^T^ (NCBI accession number NZ_SSCL00000000) and *S. susongensis* A31^T^ (NZ_SSNH00000000), *S. albida* LC13^T^ (NZ_SWDW00000000), and *S. humi* MUSC 117^T^ (NZ_JTDL00000000) were 23.20, 23.50, and 22.80%, respectively, which were below the 70% DDH species boundary as recommended by Meier-Kolthoff et al. [28]. The values of OrthoANIu, ANI-MUMmer (ANIm), and ANI-Blast (ANIb) of strain NEAU-HV1^T^ with *S. susongensis* A31^T^, *S. albida* LC13^T^, and *S. humi* MUSC 117^T^ (Table 3) were below the 94–96% cut-off value previously proposed for species delimitation [42], indicating that strain NEAU-HV1^T^ represents a putative novel species of the genus *Sinomonas*.

The whole cell sugars of strain NEAU-HV1^T^ were galactose, mannose, and rhamnose, which were consistent with type strains of *Sinomonas* [6]. The major cellular fatty acids (>5% of the total fatty acids) of strain NEAU-HV1^T^ were *anteiso*-C_15:0_ (44.6%), *anteiso*-C_17:0_ (27.0%), C_16:0_ (20.5%), and *iso*-C_15:0_ (7.9%). The fatty acid profile of strain NEAU-HV1^T^ was similar to those of closely related strains *S. susongensis* A31^T^, *S. albida* LC13^T^, and *S. humi* MUSC 117^T^, which support the placement of strain NEAU-HV1^T^ within the genus *Sinomonas*. However, some quantitative differences clearly distinguished strain NEAU-HV1^T^ from the other reference strains (Table 4). For example, C_16:0_ (20.5%) was detected as a major component in strain NEAU-HV1^T^, while it was a minor component (<5%) in the related strains. Additionally, *iso*-C_16:0_ was not detected in strain NEAU-HV1^T^, but the three reference strains had this component at 3.8, 6.8, and 11.4%, respectively. The polar lipids of strain NEAU-HV1^T^ were diphosphatidylglycerol, phosphatidylglycerol, phosphatidylinositol, and four glycolipids (Figure S3). This pattern was similar to the three related strains, which contained diphosphatidylglycerol, phosphatidylglycerol, and phosphatidylinositol as the major components [2,5,7]. The respiratory quinone system of strain NEAU-HV1^T^ consisted of MK-9(H_2_), MK-10(H_2_), and MK-8(H_2_) (approx. 89, 10, and 1%, respectively). The menaquinone composition was in agreement with the previous descriptions that MK-9(H_2_) was the predominant menaquinone of members of *Sinomonas* [2,5,7]. A comparison of the menaquinones in strain NEAU-HV1^T^ and three other related strains demonstrated that the menaquinone composition of NEAU-HV1^T^ was very similar to that of *S. susongensis* A31^T^ [5], however, the minor component MK-8(H_2_) in NEAU-HV1^T^ became the major component in *S. albida* LC13^T^ and no MK-8(H_2_) was detected in *S. humi* MUSC 117^T^ [2,7]. The DNA G+C content of strain NEAU-HV1^T^ was 67.64 mol%, which was within the range (66.6–71.8 mol%) previously obtained for species of the genus *Sinomonas* [5].

On the basis of morphological, physiological, chemotaxonomic, and phylogenetic results, strain NEAU-HV1^T^ is considered to represent a novel species within the genus *Sinomonas*, for which the name *Sinomonas gamaensis* sp. nov. is proposed.

### 3.2. Description of Sinomonas gamaensis sp. nov.

*Sinomonas gamaensis* sp. nov. (ga.ma.en’sis N.L. fem. adj. *gamaensis* of or belonging to Gama, the location of the soil sample from which the type strain was isolated).

Cells are aerobic, Gram staining positive, non-motile, bent rods at the beginning phase of growth, then fragments into coccoid shape. Colonies are circular, convex and pale yellow, with a smooth surface and diameter of 0.5–1.5 mm after incubation on NA for 48 h at 28 °C and grows at 10–45 °C (optimum, 30 °C), at pH 5–10 (optimum, pH 8), and with 0–4% (w/v) NaCl (optimum, 1%). Positive for decomposition of cellulose, nitrate reduction, urease, citrate utilization, and Voges–Proskauer reaction; but negative for H_2_S production and methyl red reaction. Aesculin is hydrolyzed, but gelatin, starch, and Tween 20, 40, and 80 are not. The following compounds are utilized as the sole source of carbon and energy: L-alanine, L-arabinose, L-arginine, D-fructose, D-galactose, lactose, raffinose, L-rhamnose, L-threonine, L-tyrosine, and D-xylose. The following compounds are not utilized as the sole source of carbon and energy: creatine, D-galactitol, L-glycine, inositol, D-mannitol. Acid is produced from D-fructose, and D-turanose; but not from N-acetylglucosamine, D-arabinose, L-arabinose, arbutin, D-cellobiose, L-fucose, D-galactose, D-glucose, D-lactose, D-maltose, D-mannose, methyl α-D-mannopyranoside, L-rhamnose, D-ribose, salicin, D-sorbitol, D-tagatose, trehalose, and D-xylose. Positive for nitrate reduction, indole production, arginine dihydrolase, urease; but negative for D-glucose fermentation. Activity is detected for esterase (C4), esterase lipase (C8), lipase (C14), valine arylamidase, α-galactosidase, N-acetyl-β-glucosaminidase, and α-mannosidase; but not for cysteine arylamidase. The whole cell sugars contain galactose, mannose, and rhamnose. The polar lipids consist of diphosphatidylglycerol, phosphatidylglycerol, phosphatidylinositol, and four glycolipids. The predominant respiratory quinone comprises MK-9(H_2_), with a minor amount of MK-10(H_2_) and MK-8(H_2_). The major cellular fatty acids (>5%) are *anteiso*-C_15:0_, *anteiso*-C_17:0_, C_16:0_, and *iso*-C_15:0_.

The type strain, NEAU-HV1^T^ (= DSM 104514^T^ = CCTCC M 2017246^T^), was isolated from a soil sample collected from Gama, Hadjer lamis, Chad. The G+C content of the type strain is 67.64 mol%, calculated from the assembly for the draft genome sequence.

### 3.3. Antifungal Activity of NEAU-HV1^T^ against E. turcicum

**Figure 4:**
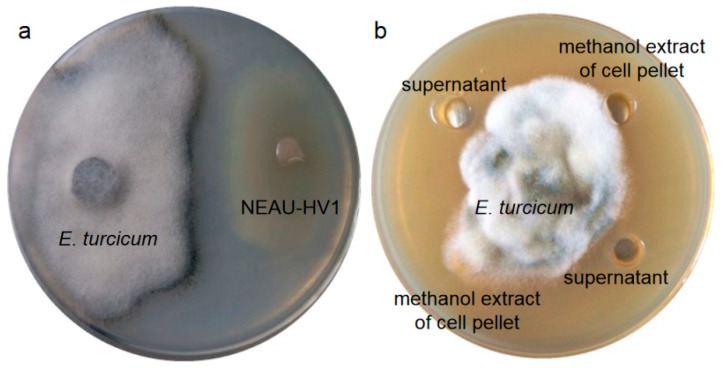
The antagonistic activity of NEAU-HV1^T^ against *E. turcicum* (**a**) and the antifungal activity of the supernatant and cell pellet of NEAU-HV1^T^ against *E. turcicum* (**b**).

**Figure 5:**
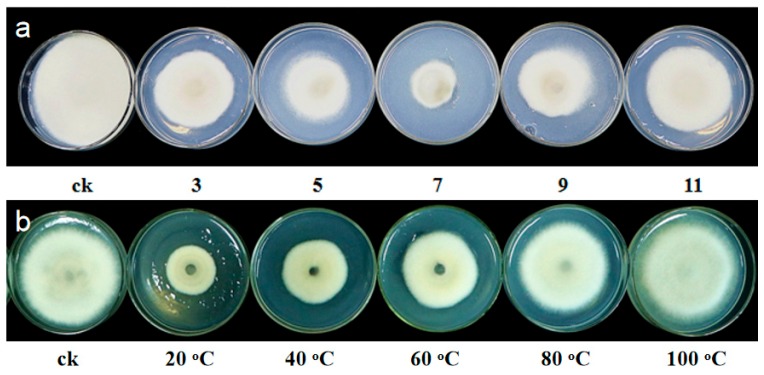
The effects of pH (**a**) and temperature (**b**) on the antifungal activity of the supernatant of NEAU-HV1^T^.

Strain NEAU-HV1^T^ possessed antagonic activity against *E. turcicum* with an inhibitory ratio of 56% to the mycelia growth, however, no inhibitory effect on the growth of five other phytopathogenic fungi was observed (Figure 4a). Comparison of the antifungal activity of the supernatant with that of the cell pellet suggested that strain NEAU-HV1^T^ could produce proteins or lipopeptides as the antifungal substances, because only the supernatant could inhibit the growth of *E. turcicum* (Figure 4b). The antifungal substances were sensitive to pH and temperature (Figure 5), which further confirmed that they were proteins. The antiSMASH analysis led to the identification of seven gene clusters, however, they showed low similarity to the known gene clusters of bottromycin, type III polyketide, two nonribosomal peptides, terpene, bacteriocin, and microansamycin. Therefore, the relationships between the corresponding secondary metabolites produced by NEAU-HV1^T^ and the antifungal activity are still ambiguous. To date, only 10 species of *Sinomonas* have been described, and *S. atrocyaneus* exhibited antimicrobial activity against *Acidovorax citrulli*, which is the pathogen of bacterial fruit blotch [12]. To the best of our knowledge, this is the first report on the antifungal activity of *Sinomonas* against plant pathogenic fungi.

## 4. Conclusions

A novel strain NEAU-HV1^T^ that exhibits antifungal activity against *E. turcicum* was isolated from a soil sample. Morphological features and phylogenetic analysis based on 16S rRNA gene sequences and genomes suggested that strain NEAU-HV1^T^ belonged to the genus *Sinomonas*. Chemotaxonomic and biochemical characteristics together with DDH relatedness values and ANI values clearly indicated that strain NEAU-HV1^T^ could be differentiated from the closely related strains *S. susongensis* A31^T^, *S. humi* MUSC 117^T^, and *S. albida* LC13^T^. Based on the polyphasic analysis, it is suggested that the strain NEAU-HV1^T^ represents a novel species of the genus *Sinomonas*, for which the name *Sinomonas gamaensis* sp. nov. is proposed. The type strain is NEAU-HV1^T^ (= DSM 104514^T^ = CCTCC M 2017246^T^). 

## Figures and Tables

**Table 1:** Differential characteristics between strain NEAU-HV1^T^ and closely related members of the genus *Sinomonas*.

Characteristic	NEAU-HV1T	*S. susongensis* A31^T^	*S. albida* LC13^T^	*S. humi* MUSC 117^T^
Nitrate reduction	+	‒	+	‒
Production of urease	+	+	‒	‒
Hydrolysis of Starch	‒	‒	+	‒
Citrate utilization	+	+	‒	+
Voges–Proskauer reaction	+	+	+	‒
Utilization of:				
L-Tyrosine	+	‒	+	‒
L-Glycine	‒	+	+	+
L-Arginine	+	‒	+	+
L-Threonine	+	‒	+	‒
L-Alanine	+	+	+	‒
Creatine	‒	+	‒	+
Raffinose	+	‒	+	+
L-Rhamnose	+	+	+	‒
D-Mannitol	‒	+	+	‒
Inositol	‒	‒	+	‒
L-Arabinose	+	+	‒	‒
D-Xylose	+	+	+	‒
Lactose	+	‒	+	‒
D-Fructose	+	‒	+	‒
D-Galactitol	‒	+	+	+
D-Galactose	+	+	+	‒
G+C mol%	67.64	68.64	69.46	67.16

All data were obtained from this study. +, positive; ‒, negative. All strains were positive for the utilization of glucose, sucrose, ribose, maltose, mannose, sorbose, L-aspartic acid, L-proline, L-glutamine, L-serine, L-asparagine, L-glutamic acid, decomposition of cellulose, and the hydrolysis of aesculin. All strains were negative for H_2_S production, liquefaction of gelatin, hydrolysis of Tweens 20, 40, and 80, and the methyl red test.

**Table 2:** General features of the genome of NEAU-HV1^T^.

Features	Values
Genome size (bp)	4,320,429
DNA G+C (%)	67.64
Number of contigs	39
Number of CDS	4182
RNA genes	46
rRNA	3
tRNA	43
N50 (bp)	233,829
N90 (bp)	55,667
Numbers of genomics islands	11
Numbers of prophage	3
Numbers of CRISPR	3

**Table 3:** The digital DNA–DNA hybridization (DDH) and average nucleotide identity (ANI) values between strain NEAU-HV1^T^ and the closely related members of the genus *Sinomonas*.

Strains	DDH (%)	OrthoANIu	ANIm	ANIb
*S. susongensis* A31^T^	23.20	80.23	85.16	79.34
*S. albida* LC13^T^	23.50	80.08	85.49	79.45
*S. humi* MUSC 117^T^	22.80	79.72	85.17	78.94

**Table 4:** Cellular fatty acid compositions (%) of strain NEAU-HV1^T^ and its closest phylogenetic neighbors.

Fatty Acid	NEAU-HV1^T^	*S. susongensis* A31^T^	*S. albidus* LC13^T^	*S. humi* MUSC 117^T^
*Iso*-C_15:0_	7.9	12.1	13.5	8.8
*Anteiso*-C_15:0_	44.6	55.2	39.2	41.4
C_16:0_	20.5	3.5	2.1	1.8
*Iso*-C_16:0_	–	2.7	7.8	12.7
*Anteiso*-C_17:0_	26.9	22.8	29.4	16.2
*Iso*-C_17:0_	–	2.6	5.9	1.3
C_18:1_ω7c	–	–	–	16.2

–, Not detected. Fatty acids found in amounts <1.0% in the strains are not shown. All data were obtained from this study.

## Supplementary Materials

The following are available online at https://www.mdpi.com/2076-2607/7/6/170/s1, Figure S1: Maximum likelihood tree based on 16S rRNA gene sequences showing relationship between strain NEAU-HV1^T^ and related taxa. Only bootstrap values above 50% (percentages of 1000 replications) are indicated. Bar, 0.02 nucleotide substitutions per site, Figure S2: Minimum evolution tree based on 16S rRNA gene sequences showing relationship between strain NEAU-HV1^T^ and related taxa. Only bootstrap values above 50% (percentages of 1000 replications) are indicated, Figure S3: The polar lipids of strain NEAU-HV1^T^ after two-dimensional TLC and sprayed with molybdophosphoric acid. a, using molybdenum blue reagent; b, using ninhydrin reagent; c, using anisaldehyde reagent, d, using molybdophosphoric acid reagent. Diphosphatidylglycerol (DPG), phosphatidylglycerol (PG), phosphatidylinositol (PI) and glycolipids 1–4 (GL1–4), Table S1: Differential characteristics between strain NEAU-HV1^T^ and the closely related memebers of the genus *Sinomonas* in API tests.
