# Supplementary material for: Characterization of Sinomonas gamaensis sp. nov., a Novel Soil Bacterium with Antifungal Activity against Exserohilum turcicum
Source: Microorganisms. 2019 Jun 8;7(6):170. doi: 10.3390/microorganisms7060170 (PMC6617354; doi:10.3390/microorganisms7060170)
Supplement: Supplementary file 1 [file microorganisms-07-00170-s001.pdf]

microorganisms

MDPI

Supplementary Materials

Characterization of *Sinomonas gamaensis* sp. nov., a Novel Soil Bacterium with Antifungal Activity against *Exserohilum turcicum*

Yansong Fu [1], Rui Yan [1], Dongli Liu [1], Junwei Zhao [1], Jia Song [1], Xiangjing Wang [1], Lin Cui [1], Ji Zhang [1,*] and Wensheng Xiang [1,2,*]

1 Heilongjiang Provincial Key Laboratory of Agricultural Microbiology, Northeast Agricultural University, Harbin 150030, China; yansongfu1@163.com (Y.F.); 15546073308@163.com (Y.R.); 17862181612@163.com (D.L.); guyan2080@126.com (J.Z.); songjia-hit@hotmail.com (J.S.); wangneau2013@163.com (X.W.); 15663843306@163.com (L.C.)

2 State Key Laboratory for Biology of Plant Diseases and Insect Pests, Institute of Plant Protection, Chinese Academy of Agricultural Sciences, Beijing 100193, China

* Correspondence: zhangji@neau.edu.cn (J.Z.); xiangwensheng@neau.edu.cn (W.X.)

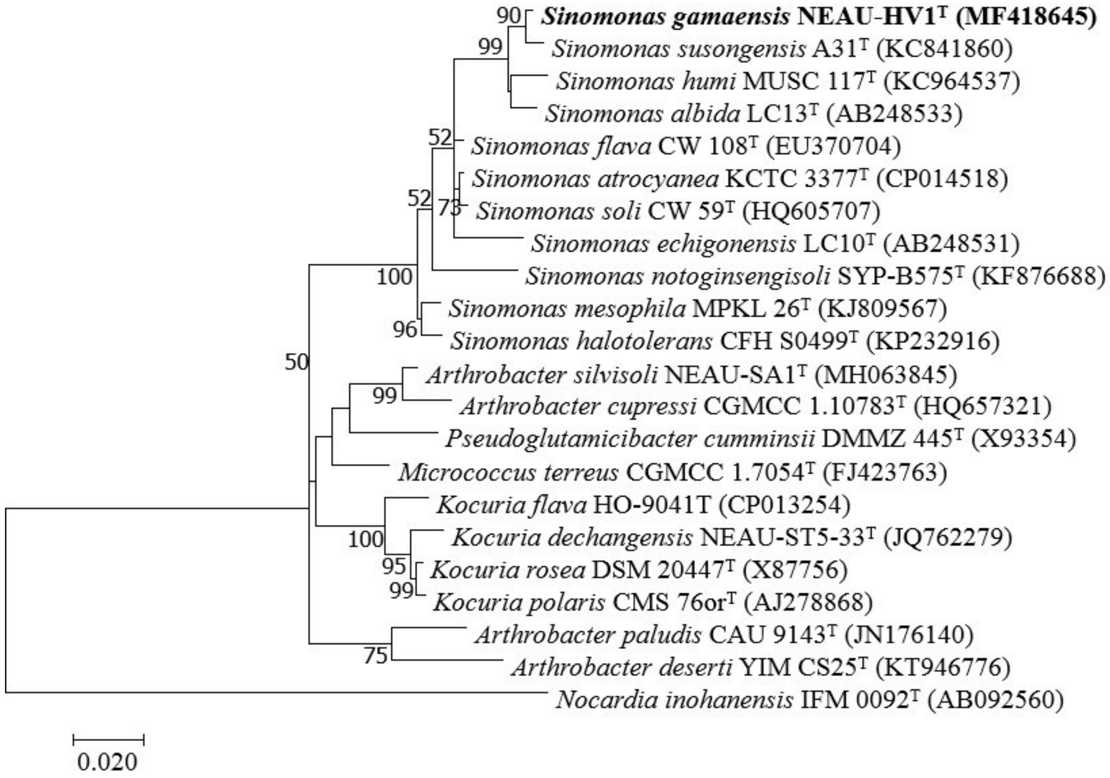

Figure S1. Maximum likelihood tree based on 16S rRNA gene sequences showing relationship between strain NEAU-HV1^T and related taxa. Only bootstrap values above 50% (percentages of 1000 replications) are indicated. Bar, 0.02 nucleotide substitutions per site.

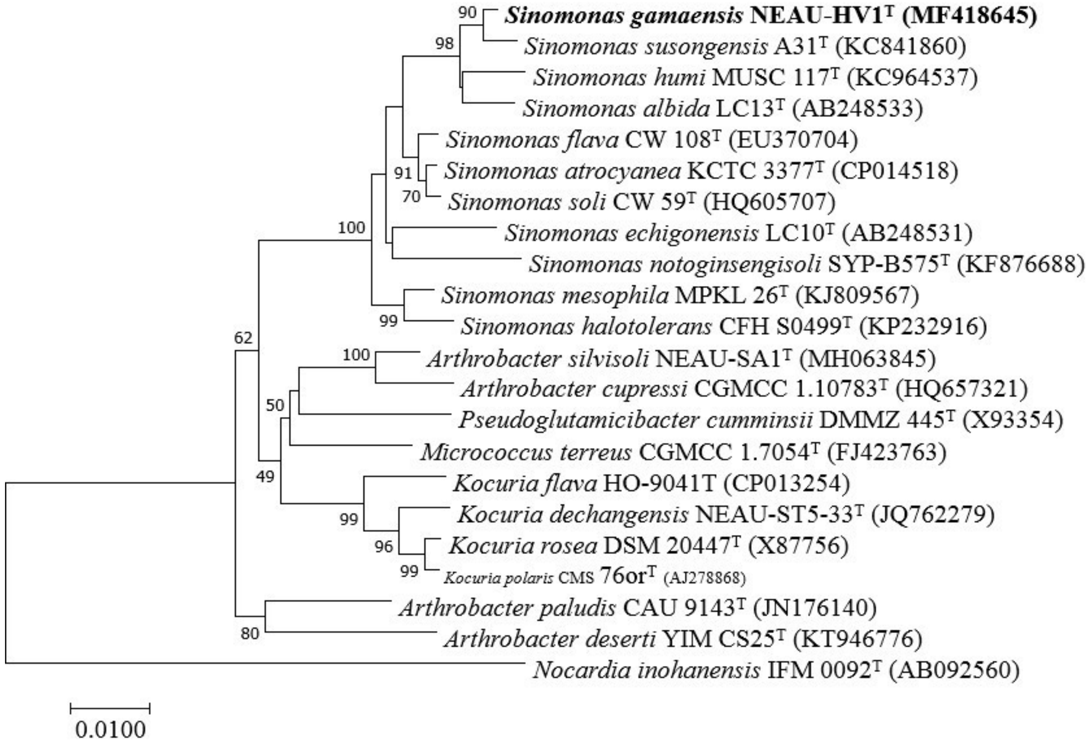

Figure S2. Minimum evolution tree based on 16S rRNA gene sequences showing relationship between strain NEAU-HV1^T and related taxa. Only bootstrap values above 50% (percentages of 1000 replications) are indicated.

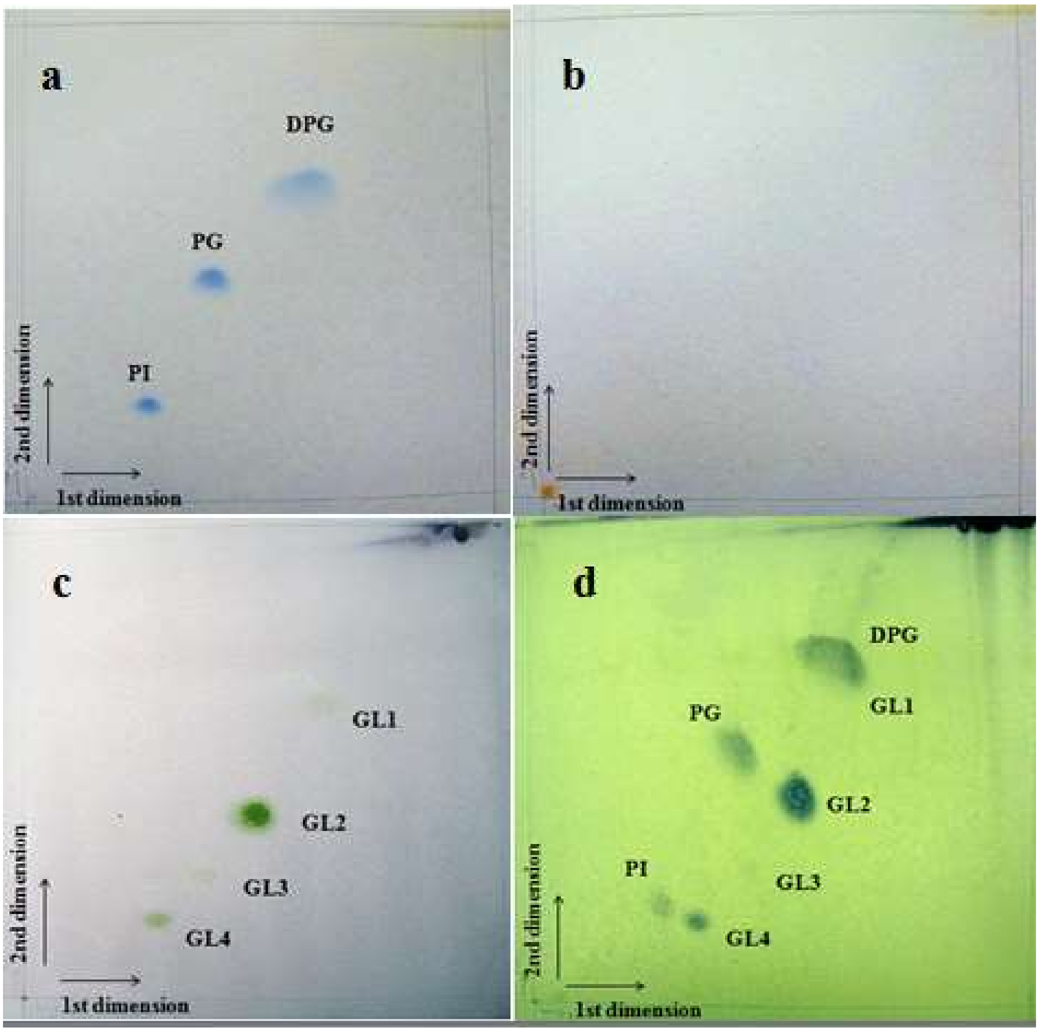

Figure S3. The polar lipids of strain NEAU-HV1ᵀ after two-dimensional TLC and sprayed with molybdophosphoric acid. (**a**) Using molybdenum blue reagent; (**b**) using ninhydrin reagent; (**c**) using anisaldehyde reagent, (**d**) using molybdophosphoric acid reagent. Diphosphatidylglycerol (DPG), phosphatidylglycerol (PG), phosphatidylinositol (PI) and glycolipids 1–4 (GL1–4).

Table S1. Differential characteristics between strain NEAU-HV1^T and the closely related memebers of the genus *Sinomonas* in API tests.

Characteristic	NEAU-HV1^T	*S. susongensis* A31^T	*S. albida* LC13^T	*S. humi* MUSC 117^T
Acid production from (API 50CH)				
D-arabinose	−	+	−	−
L-arabinose	−	+	+	−
D-ribose	−	+	+	−
D-xylose	−	+	+	−
D-galactose	−	+	+	−
D-fructose	+	−	−	+
D-mannose	−	+	+	+
L-rhamnose	−	+	+	−
D-sorbitol	−	+	−	−
Methyl α-D-mannopyranoside	−	−	+	−
N-Acetylglucosamine	−	+	+	−
Arbutin	−	+	+	−
Salicin	−	+	+	−
D-Cellobiose	−	+	+	−
D-Maltose	−	+	+	−
D-Lactose	−	−	+	−

Trehalose	–	–	+	–
D-glucose	–	+	+	+
D-Turanose	+	–	–	+
D-Tagatose	–	+	+	–
L-Fucose	–	–	+	–
Biochemical characteristics (API 20NE)				
Nitrate reduction	+	–	–	+
Indole production	+	–	–	–
D-glucose fermentation	–	+	–	–
Arginine dihydrolase	+	–	–	+
Urease	+	+	–	–
Enzyme activities (API ZYM)				
Esterase (C4)	+	–	+	+
Esterase lipase (C8)	+	–	–	+
Lipase (C14)	+	–	–	+
Valine arylamidase	+	–	–	+
Cysteine arylamidase	–	–	–	+
α-Galactosidase	+	+	–	+
N-Acetyl-β-glucosaminidase	+	–	+	+
α-Mannosidase	+	+	–	+

All data were obtained from this study. +, positive; –, negative. In API 50 CH test, all strains are positive for glycerol, D-mannitol, esculin ferric citrate; but negative for erytritol, L-xylose, D-adonitol, methyl β-D-xylopyranoside, L-sorbose, dulctiol, inositol, Methyl α-D-glucopyranoside, amygdalin, D-Melibiose, D-Sucrose, inulin, D-melezitose, D-raffinose, starch, glycogen, xylitol, gentiobiose, D-lyxose, D-fucose, D-arabitol, L-arabitol, potassium gluconate, potassium 2-ketogluconate, potassium 5-ketogluconate. In API ZYM test, all strains are positive for leucine arylamidase, acid phosphatase, naphthol-AS-BI-phosphohydrolase, β-galactosidase, α-glucosidase, and β-glucosidase; but negative for alkaline phosphatase, trypsin, α-chymotrypsin, β-glucuronidase, and β-fucosidase.

© 2019 by the authors. Submitted for possible open access publication under the terms and conditions of the Creative Commons Attribution (CC BY) license (http://creativecommons.org/licenses/by/4.0/).
